# Association between TNIP1, MPHOSPH6 and ZNF208 genetic polymorphisms and the coronary artery disease risk in Chinese Han population

**DOI:** 10.18632/oncotarget.20432

**Published:** 2017-08-24

**Authors:** Yanbin Song, Mengdan Yan, Jing Li, Jingjie Li, Tianbo Jin, Chao Chen

**Affiliations:** ^1^ Key Laboratory of Resource Biology and Biotechnology in Western China (Northwest University), Ministry of Education, Xi’an, Shaanxi 710069, China; ^2^ School of Life Sciences, Northwest University, Xi’an, Shaanxi 710069, China; ^3^ Department of Cardiovascular, Yanan University Affiliated Hospital, Yanan, Shaanxi 716000, China

**Keywords:** coronary artery disease (CAD), polymorphisms, ZNF208, TNIP1, MPHOSPH6

## Abstract

**Introduction:**

Coronary artery disease (CAD) is a common disease and among the leading cause of death in the general population. Inherited factors are involved in the pathogenesis of CAD.

**Aims:**

Our study examined whether SNPs in TNIP1, MPHOSPH6, ZNF208 to be associated with CAD risk in a Chinese Han population. We recruited 596 CAD patients, 603 controls and genotyping fifteen SNPs using Sequenom MassARRAY. For association analysis between TNIP1, MPHOSPH6 and ZNF208 and CAD was determined by Odds ratios (ORs) with 95% confidence intervals (CIs) using Logistic Regression.

**Results:**

The results indicated in allel model, the rs960709 in TNIP1 was associated with CAD risk (OR = 0.78, 95%CI = 0.65-0.94, P=0.010). The genetic model results showed that the rs960709 (A/G) polymorphism was associated with the risk of developing CAD in codominant, Dominant and Log-additive. The rs1056654 A/A allele and CAD patients compared to the healthy controls in recessive model (OR = 0.55, 95%CI = 0.34-0.90; P = 0.018). We also found that three SNPS in ZNF208 associated with CAD, respectively, rs2188971, rs8103163 and rs7248488.

Linkage disequilibrium (LD) and haplotype analyses of the SNPs found that the CTA haplotype (rs1056675, rs1056654, rs11859599) and rs2188972A/rs2188971T/rs8103163A/rs7248488A (ATAA) were associated with CAD.

**Conclusion:**

In conclusion, the present study provided evidence that SNPs in the TNIP1, ZNF208 and MPHOSPH6 were associated with CAD in Chinese Han population. It is possible that these SNPs are CAD risk factors and these data can provide.

## INTRODUCTION

Coronary artery disease (CAD) is a common disease, and among the leading cause of death in the general population [1]. It is the most common form of heart disease known that as a complex disease. There are many risk factors for coronary heart disease, including alcoholism, family history, hypertension, diabetes mellitus and hyperlipidemia [2]. It is generally believed that CAD and its related risk factors are largely genetic controlled [3]. Whereas only few of individuals with the risk factors of CAD eventually develop this disease. Therefore, inherited factors participate in the pathogenesis of CAD [4]. Telomeric DNA is located at the end of chromosomes, consists of TTAGGG tandem repeats, although the full extent of their functions is not fully understood and is important for maintaining genomic stability.

CAD is an age related disease. In humans, telomere shortening is regarded as a predictor of aging and aging-related diseases [5]. Many previous studies have suggested that a relatively short telomere length (TL) is associated with a higher risk of developing aging-related chronic diseases, particularly cardiovascular dysfunctions [6–9]. Observationally, short telomeres are associated with an increased risk of ischemic heart disease [8, 10, 11]. Genetic variants implicated in TL have been reported to be associated with the incidence of aging-related chronic diseases [12]. For example, the study found rs10786775 C > G, rs11591710 A > C single nucleotide polymorphisms (SNPs) in OBFC1 were associated with a higher risk of developing coronary heart CAD, and rs12696304 C > G, rs10936601 G > T, rs16847897 G > C SNPs in TERC were correlated with a higher risk of CAD and the type II diabetes mellitus (T2DM) [13]. It was also reported that genetic variations in TERT were significantly correlated with CAD in a Japanese case study [14]. Individual differences in telomere length in rodents [15, 16] and humans [17–20] suggest that this parameter is genetically determined. While evidence for the causal role of TL variation in some chronic diseases has accumulated [21], the link between TNIP1, MPHOSPH6, ZNF208 SNPs and CAD risk, has not been extensively studied in Chinese populations. In the study, we examined whether SNPs in TNIP1, MPHOSPH6, ZNF208 to be associated with CAD risk in a Chinese Han population.

## RESULTS

The comparison of basic characteristics data of the controls and CAD groups is given in Table 1. Age and sex difference were statistically significant. Table 3 summarizes the distributions of SNPs genotypes, allele frequencies and the basic information for two groups. The distribution of rs4958881 (TNIP1), rs1056629 (MPHOSPH6) in control subjects was not in the Hardy-Weinberg equilibrium and the rest of the SNP distribution complied with the Hardy–Weinberg equilibrium. In allel model, we found the rs960709 in TNIP1 was associated with CAD risk, and had a 0.78 fold risk of CAD (OR = 0.78, 95%CI = 0.65-0.94, P=0.010).

**Table 1:** Characteristics of cases and controls in this study

Variables	Cases (N)	Controls (N)	*p*
Subjects	596	603	
Gender			< 0.001
Female	220	134	
Male	376	469	
Age ± SD	61.44 ± 11.16	48.24 ± 13.05	< 0.001

*p* value ≤ 0.05 indicates statistical significance.

**Table 2:** PCR primers

SNP	1st-PCR primer sequences	2st-PCR primer sequences	UEP sequences
rs3792792	ACGTTGGATGCTCAGATCAGTTCACTCCTC	ACGTTGGATGATGGCAGCTGTTACGGCCAC	ccctTTACGGCCACCACCAAGCATG
rs4958881	ACGTTGGATGCACAAATATGTGGACAGTTT	ACGTTGGATGTGCAATTCCACCCAAGGATG	GGATGAAAGGAAGTGAGA
rs7708392	ACGTTGGATGAGGCCAACTGGTCAATTCTC	ACGTTGGATGGGGTCTCTTCTGGAACTTAG	ggggaTGGAACTTAGTAGACTAGTCA
rs10036748	ACGTTGGATGGCAAAGCAGCCCCTTTTTTC	ACGTTGGATGCTTTCATAGCATGATACACG	ACGTATGAGAAAAATAAAATAGTAA
rs960709	ACGTTGGATGTATGGGTCTTTTCAGCTCGG	ACGTTGGATGTAAGCCAGAGCTGGAGCTCA	atAGTTCCGTCCAGGGC
rs1056675	ACGTTGGATGAATACTTAAGGCTGGAGAGG	ACGTTGGATGGTCAAGCCAATTCGTACATAC	ggtgCGTACATACAATTTGGAATCAA
rs1056654	ACGTTGGATGGTATGTACGAATTGGCTTGAC	ACGTTGGATGCAGTCACTGACCTTGAATTG	ACCTTGAATTGACTTACATAAA
rs1056629	ACGTTGGATGTTTTTAGCCCCTGATCTAC	ACGTTGGATGGGTCAGTGACTGGAGAACTA	cGGAAGCAGCCCTGTAACAA
rs3751862	ACGTTGGATGTGGTGTCTCTATAGTTATT	ACGTTGGATGCATCTGTTTCAAAAACAGC	TGTTTCTAAAATGATAATCTCTTTACA
rs11859599	ACGTTGGATGTAAGAGAAGGCCGATCACAG	ACGTTGGATGCCCAGGAATGCTCCTCTTAC	CCTCTTACCCCACAGT
rs2967361	ACGTTGGATGTTACTGGGAACCAGCTTACG	ACGTTGGATGAGCTGTACCCTGACTGCTTC	tCCTGACTGCTTCTGTGTAC
rs2188972	ACGTTGGATGGGCTTGATTGGTCAAATGGC	ACGTTGGATGATTCAGAACCTGTGCAAAGC	GACTTCTCAAAGAACTAGAAA
rs2188971	ACGTTGGATGCTCTTCAAAGATCTACTTC	ACGTTGGATGCACTAAATCAGACTGCTGAG	TCCAAAACTAAAGTTGGCAAAA
rs8103163	ACGTTGGATGCCAGAAGATCTGAGATAAAG	ACGTTGGATGTTTTGGGCCAAAAACTTTG	cctGCCAAAAACTTTGGCATACT
rs7248488	ACGTTGGATGGTTCTCCAGGAACACTTATG	ACGTTGGATGGCAGAGTGTTTTCCTGGTTG	GTCATGATGAGAAGGGT

**Table 3:** Basic information of candidate SNPs in this study

SNP	Chr	Allel(A^a^/B)	Gene	MAF(Case)	MAF(Control)	HWE(P)	OR	95%CI	P
rs3792792	5q33.1	C/T	TNIP1	0.0595638	0.064676617	0.302	0.92	0.66-1.28	0.604
rs4958881	5q33.1	C/T	TNIP1	0.0713087	0.105982906	5.33E-74	0.65	0.49-0.86	0.003
rs7708392	5q33.1	G/C	TNIP1	0.2508389	0.233001658	0.820	1.10	0.91-1.33	0.308
rs10036748	5q33.1	C/T	TNIP1	0.2516779	0.233001658	0.820	1.11	0.92-1.33	0.286
rs960709	5q33.1	A/G	TNIP1	0.2533898	0.301886792	0.304	0.78	0.65-0.94	0.010
rs1056675	16q23.3	C/T	MPHOSPH6	0.4253356	0.430116473	1.000	0.98	0.83-1.15	0.813
rs1056654	16q23.3	A/G	MPHOSPH6	0.272651	0.290697674	0.323	0.91	0.77-1.09	0.326
rs1056629	16q23.3	C/T	MPHOSPH6	0.272651	0.266221374	2.39E-08	1.03	0.86-1.25	0.732
rs3751862	16q23.3	C/A	MPHOSPH6	0.0564924	0.042358804	0.287	1.35	0.93-1.97	0.111
rs11859599	16q23.3	C/G	MPHOSPH6	0.2432886	0.23880597	0.093	1.02	0.85-1.24	0.797
rs2967361	16q23.3	T/G	MPHOSPH6	0.2567114	0.23255814	0.425	1.14	0.95-1.37	0.169
rs2188972	19p12	G/A	ZNF208	0.5184564	0.487562189	0.415	1.13	0.96-1.33	0.130
rs2188971	19p12	T/C	ZNF208	0.329698	0.302325581	0.700	1.14	0.96-1.35	0.150
rs8103163	19p12	A/C	ZNF208	0.3288591	0.3026534	0.772	1.13	0.95-1.34	0.167
rs7248488	19p12	A/C	ZNF208	0.329698	0.303482587	0.631	1.13	0.95-1.34	0.168

^a^ Minor allele; MAF, minor allelic frequency; HWE, Hardy-Weinberg equilibrium; ORs, odds ratios; CI: confidence interval.

HWE *p*-value ≤ 0.05 was excluded; *p* value ≤ 0.05 indicates statistical significance.

Table 4 shows the genetic models adjusted for age, sex, and smoking. The rs960709 (A/G) polymorphism was positively associated with the risk of CAD in codominant (OR = 0.55, 95%CI = 0.32-0.80; P = 8*10^-4^), Dominant (OR = 0.59, 95%CI = 0.45-0.78; P = 2*10^-4^), Log-additive (OR = 0.67, 95%CI = 0.54-0.84; P = 4*10^-4^). There was significant association between the rs1056654 A/A allele and CAD patients compared to the healthy controls in recessive model (OR = 0.55, 95%CI = 0.34-0.90; P = 0.018). We also found that three SNPS in ZNF208 associated with CAD, respectively, rs2188971 (dominant: OR = 1.31, 95%CI = 1.01-1.70, P = 0.04; Log additive: OR = 1.23, 95%CI = 1.01-1.50, P = 0.038); rs8103163(dominant: OR = 1.31, 95%CI = 1.01-1.69, P = 0.044; Log additive: OR = 1.23, 95%CI = 1.01-1.50, P = 0.044); rs7248488(dominant: OR = 1.31, 95%CI = 1.01-1.69, P = 0.044).

**Table 4:** Association between significant SNPs and risk of CAD in genetics models

SNP	Model	Genotype	control	case	OR (95% CI)	P-value
rs960709	Codominant	G/G	253 (47.7%)	326 (55.2%)	1	8.00E-04
		A/G	234 (44.1%)	229 (38.8%)	0.60 (0.45-0.80)	
		A/A	43 (8.1%)	35 (5.9%)	0.55 (0.32-0.96)	
	Dominant	G/G	253 (47.7%)	326 (55.2%)	1	2.00E-04
		A/G-A/A	277 (52.3%)	264 (44.8%)	0.59 (0.45-0.78)	
	Recessive	G/G-A/G	487 (91.9%)	555 (94.1%)	1	0.19
		A/A	43 (8.1%)	35 (5.9%)	0.70 (0.41-1.20)	
	Log-additive	—	—	—	0.67 (0.54-0.84)	4.00E-04
rs1056654	Codominant	G/G	308 (51.2%)	307 (51.5%)	1	0.059
		A/G	238 (39.5%)	253 (42.5%)	0.99 (0.76-1.30)	
		A/A	56 (9.3%)	36 (6%)	0.55 (0.33-0.92)	
	Dominant	G/G	308 (51.2%)	307 (51.5%)	1	0.46
		A/G-A/A	294 (48.8%)	289 (48.5%)	0.91 (0.70-1.17)	
	Recessive	G/G-A/G	546 (90.7%)	560 (94%)	1	0.018
		A/A	56 (9.3%)	36 (6%)	0.55 (0.34-0.90)	
	Log-additive	—	—	—	0.85 (0.69-1.04)	0.11
rs2188971	Codominant	C/C	295 (49%)	268 (45%)	1	0.11
		T/C	250 (41.5%)	263 (44.1%)	1.28 (0.98-1.69)	
		T/T	57 (9.5%)	65 (10.9%)	1.45 (0.91-2.29)	
	Dominant	C/C	295 (49%)	268 (45%)	1	0.04
		T/C-T/T	307 (51%)	328 (55%)	1.31 (1.01-1.70)	
	Recessive	C/C-T/C	545 (90.5%)	531 (89.1%)	1	0.26
		T/T	57 (9.5%)	65 (10.9%)	1.28 (0.83-1.99)	
	Log-additive	—	—	—	1.23 (1.01-1.50)	0.038
rs8103163	Codominant	C/C	295 (48.9%)	268 (45%)	1	0.12
		A/C	251 (41.6%)	264 (44.3%)	1.28 (0.97-1.68)	
		A/A	57 (9.4%)	64 (10.7%)	1.42 (0.90-2.26)	
	Dominant	C/C	295 (48.9%)	268 (45%)	1	0.044
		A/C-A/A	308 (51.1%)	328 (55%)	1.31 (1.01-1.69)	
	Recessive	C/C-A/C	546 (90.5%)	532 (89.3%)	1	0.29
		A/A	57 (9.4%)	64 (10.7%)	1.27 (0.82-1.97)	
	Log-additive	—	—	—	1.23 (1.01-1.50)	0.044
rs7248488	Codominant	C/C	295 (48.9%)	268 (45%)	1	0.12
		A/C	250 (41.5%)	263 (44.1%)	1.28 (0.98-1.69)	
		A/A	58 (9.6%)	65 (10.9%)	1.40 (0.89-2.21)	
	Dominant	C/C	295 (48.9%)	268 (45%)	1	0.044
		A/C-A/A	308 (51.1%)	328 (55%)	1.31 (1.01-1.69)	
	Recessive	C/C-A/C	545 (90.4%)	531 (89.1%)	1	0.32
		A/A	58 (9.6%)	65 (10.9%)	1.25 (0.81-1.93)	
	Log-additive	—	—	—	1.22 (1.00-1.49)	0.047

ORs, odds ratios; CI: confidence interval.*p value ≤ 0.05 indicates statistical significance.

**Figure 1:**
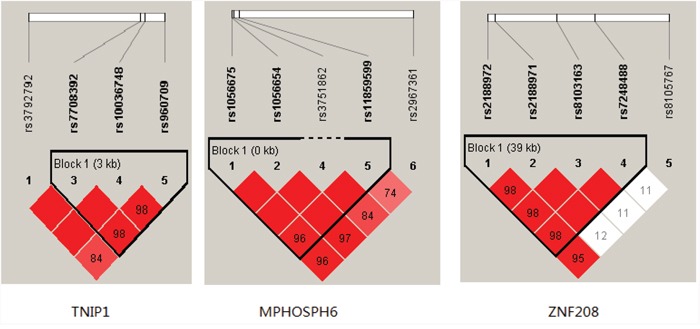
Haplotype block map for the TNIP1, MPHOSPH6 and ZNF208 SNPs genotyped in this study

Linkage disequilibrium (LD) and haplotype analysis of the SNPs in case and control samples were further investigated. The association between the TNIP1 haplotype and the risk of CAD was shown in Table 5a. The study found that the CTA haplotype (rs1056675, rs1056654, rs11859599) was associated with CAD and may be a risk factor for CAD after adjustment (OR = 1.57; 95% CI, 1.02–2.40; p =0.040). The LD between the five SNPs were observed (Figure 1). The ZNF208 haplotype and the risk of CAD are listed in Table 5b. Haplotype estimation analysis showed that the haplotype have a significant increased risk of CAD was found in the rs2188972A/rs2188971T/rs8103163A/rs7248488A (ATAA) genotypes compared with the GCCC genotype (OR=1.26, 95%CI=1.02-1.55; P=0.034).

**Table 5A:** Haplotype frequencies and their associations with CAD risk in ZNF208

	Rs2188972	rs2188971	rs8103163	rs7248488	Freq	OR (95% CI)	P-value
1	G	C	C	C	0.495	1	—
2	A	T	A	A	0.314	1.26 (1.02 - 1.55)	0.034
3	A	C	C	C	0.188	1.09 (0.85 - 1.39)	0.520

ORs, odds ratios; CI: confidence interval.*p value ≤ 0.05 indicates statistical significance.

**Table 5B:** Haplotype frequencies and their associations with CAD risk in MPHOSPH6

	rs1056675	rs1056654	rs11859599	Freq	OR (95% CI)	P-value
1	C	G	G	0.427	1	—
2	T	A	G	0.282	0.87 (0.70 - 1.09)	0.230
3	T	G	C	0.239	1.03 (0.82 - 1.30)	0.780
4	T	G	G	0.051	1.57 (1.02 - 2.40)	0.040

ORs, odds ratios; CI: confidence interval.*p value ≤ 0.05 indicates statistical significance.

## DISCUSSION

We performed this case-control study to assess the correlation between 15 SNPs in TNIP1, MPHOSPH6 and ZNF208 and the risk of CAD in Chinese Han population. Our results suggested that rs960709 (A) in TNIP1 and rs1065654 (A) in MPHOSPH6 were associated with a decreased risk of CAD. In addition in ZNF208 gene, we found three SNPs (rs2188971, rs8103163, rs7248488) were correlated with a higher risk of CAD.

The ZNF208 gene is located on the human chromosome 19p12. ZNF is the function of the largest human gene family mediated by DNA, RNA, protein interactions, and regulation of gene expression by interacting with other proteins or lipids with a special DNA sequence [22]. They can modulate the expression of genes as a potent transcriptional repressor [23, 24]. To date, only a small number of studies have initiated related studies in ZNF208 regulation of gene expression, with respect to the progression and treatment of several diseases. In recent years, the genome-wide association study (GWAS) of rs8105767 in ZNF208 has been described in relation to telomere length. It was identified to be related to a shortened telomere length and CAD in a European population [25]. Few scholars have studied the association between ZNF208 and CAD until now. The results of this study study suggested that rs2188971, rs8103163 and rs7248488 were indeed associated with the CAD risk. These variants may play a role in regulating gene activity and expression of mRNA, or shortening telomere length and participating in the development of disease.

Transcription factor kappa B (NF-κB) is an important regulator of many physiological and pathophysiological processes. It is a transcription factor and plays an important role in inflammation and immune response, cell proliferation and apoptosis. In recent years more attention has been given to the relationship between NF-κB and CAD [26, 27]. Nevertheless NF-κB activity is tightly controlled by several regulatory proteins, such as TNIP1 (ABIN-1) which can inhibit the NF-κB activation induced by tumor necrosis factor, interleukin-1, EGF and lipopolysaccharide [28, 29]. Loss of protein A20, which binds with the protein (ABIN-1) encoded by the gene TNF α-induced protein 3-interacting protein 1 (TNIP1), resulted in inflammation and autoimmune. Naveed Akbar, et al reported that ABIN-1 was associated with cardiovascular disease in mice [30]. However, present studies mostly focused on the associations between TNIP1 gene polymorphisms and systemic lupus erythematosus [31]. However, up to now, there were fewer reports on the association between TINP1 polymorphisms and CAD risk. Our results indicated rs960709 in TNIP1were found to be associated with the risk of developing CAD.

MPHOSPH6 play an important role in the recruitment of the exosome to the pre-rRNA [32]. MPHOSPH6 may also be involved in regulating shrimp cell cycle and ovary development [33]. MPHOSPH6 is located on chromosome 16q23.3 and encodes the M-phase phosphoprotein 6 (MPP6) that is important for the maturation of 5.8S rRNA. MPP6 is also a RNA-binding protein, which preferentially binds to pyrimidine homopolymers [34].

In our study, we detected five SNPs of *MPHOSPH6*, and found that genetic polymorphisms of MPHOSPH6 (rs1056654) is associated with CAD, which may shed a new light on the in-depth study for this gene.

In conclusion, the present study provided evidence that SNPs in the TNIP1, ZNF208 and MPHOSPH6 were associated with CAD in Chinese Han population. It is possible that these SNPs are CAD risk factors and these data can provide. While the development of CAD and the relationship between telomerase genes is worth further research.

## MATERIALS AND METHODS

### Subjects

A total of 596 CAD patients, 603 controls were enrolled following institutional ethical approval by Yanan University Affiliated Hospital. All CAD patients were diagnosed according to ischemic heart disease diagnostic criteria issued by WHO in 1979 or the presence of stenosis of more than 50% luminal diameter in at least one significant coronary artery on coronary angiography angiography. All controls were individuals free of CAD. All these CAD patients and controls were unrelated Chinese Han people. All participants were interviewed, and data on hypertension, diabetes mellitus, dyslipidemia, medical history including family history, smoking status and duration of CAD were recorded. Baseline clinical characteristics of CAD patients enrolled in this study are summarized in Table 1. Genomic DNA was extracted from the whole blood sample of each patient and control.

### Selection of single nucleotide polymorphisms and genotyping

In this study, fifteen SNPs in TNIP1, MPHOSPH6 and ZNF208 were selected from DbSNP database ( http://www.hapmap.org/index.html.en) and SNP Consortium database ( http://snp.cshl.org/) for analysis and each had minor allele frequency (MAF) of > 5% in Chinese Han population. DNA was isolated from Whole blood were used the GoldMag-Mini Whole Blood Genomic DNA Purification Kit (GoldMag Co. Ltd. Xi’an City, China) extracted. Genotypes for SNPs were determined by Sequenom MassARRAY. We used a NanoDrop 2000 (Gene Company Limited) were measured DNA concentrations. We used Sequenom MassARRAY Assay Design 3.0 Software to design a Multiplexed SNP MassEXTEND assay [35]. The PCR primers for each SNP are shown in Table 2. Data management and analysis was performed using the Sequenom Typer 4.0 Software [35, 36].

### Statistical analysis

Hardy-Weinberg equilibrium was tested using a χ^2^ test to judge the reliability of the gene frequency. Categorical data were analyzed using the chi-square test. χ^2^ test was performed to assess the distribution of sex, age of CAD among the groups. Allele and genotype frequencies were calculated using the Fisher's exact and Chi square tests, respectively. For association analysis between TNIP1, MPHOSPH6 and ZNF208 and CAD was determined by Odds ratios (ORs) with 95% confidence intervals (CIs) using Logistic Regression. Multivariate Logistic regression analysis was used to examine the association of clinical and laboratory parameters with different genotype of the SNPs polymorphism with CAD. P values were used to describe significance. Statistical significance was set as a P value<0.05. All statistical calculations were done using Statistical Package for Social Sciences (SPSS for Windows) software (version 22.0, SPSSInc., Chicago, IL, USA).
